# Relationship between epistasis and aggressiveness in resistance of pepper (*Capsicum annuum* L.) to *Phytophthora nicotianae*

**DOI:** 10.1590/S1415-47572010005000027

**Published:** 2010-06-01

**Authors:** Fethi Bnejdi, Morad Saadoun, Mohamed Bechir Allagui, Colin Hanbury, Mohamed El Gazzah

**Affiliations:** 1 Laboratoire de Génétique et Biométrie, Faculté des Sciences de Tunis, Université Tunis El ManarTunisia; 2 , Institut National de la Recherche Agronomique de TunisieTunisia; 3 Department of Agriculture and Food, Western Australia, PerthAustralia

**Keywords:** additive model, best fit model, gene effect, heredity

## Abstract

This study evaluated the types of gene action governing the inheritance of resistance to *Phytophthora nicotianae* necrosis in populations derived from two crosses involving two susceptible (Beldi and Nabeul II) and one resistant (CM334) cultivars of pepper (*Capsicum annuum* L.). Populations, composed of Pr, Ps, F_1_ , F _2_ , BC _1_ Pr, and BC _1_ Ps generations, were inoculated with six *P. nicotianae* isolates. Generation means analysis indicated that an additive-dominance model was appropriate for *P. nicotianae* isolates Pn _Ko1_ , Pn _Ko2_ and Pn _Kr1_ , which showed low aggressiveness in the two crosses. For the more aggressive isolates Pn _Bz1_ , Pn _Bz2_ and Pn _Kr2_ , epistasis was an integral component of resistance in the two crosses. The presence of epistasis in the resistance of pepper to *P*. *nicotianae* was dependent on the level of aggressiveness of the isolates. Selection in pepper with less aggressive isolates was efficient, but not with more aggressive isolates; on the other hand, selection with more aggressive isolates was more stable. The minimum number of genes controlling resistance was estimated at up to 2.71. In the majority of cases, the additive variance was significant and greater than the environmental and dominance variance.

## Introduction

One of the main factors limiting greater yields of pepper (*Capsicum annuum* L.) in Tunisia is the root necrosis *Phytophthora nicotianae*. This pathogen causes many symptoms such as wilting, necrosis and root rot. The environmental problem caused by the continuous use of chemicals for control makes breeding for resistance increasingly important. Resistance of pepper to *Phytophthora* spp. is inherited quantitatively and depends on both genetic factors and environment (Reifschneider *et al.*, 1992).

Although epistasis is common in gene systems that determine quantitative traits, it is also a major problem in studies of these traits, because it complicates the interpretation of genetic experiments and makes predictions difficult. The importance of epistasis is not well understood, and its contribution to quantitative variation was once considered to be small (Crow, 1987). Epistasis effects commonly occur in plant resistance to pests or diseases. Examples are pepper and *P. nicotianae* (Bnejdi *et al.*, 2009), pepper and *P. capsici* (Bartual *et al.*, 1993), common bean and anthracnose (Marcial and Pastor, 1994), barley and Fusarium head blight (Flavio *et al.*, 2003). There is a lack of knowledge on the contribution of pathogen aggressiveness in determining the mode of gene action. Several studies have reported that the nature and magnitude of gene action in resistance to pest and disease were determined by pathogen aggressiveness. Bartual *et al.* (1991, 1993) reported that the relative importance of higher-order epistasis in additive x additive epistasis seemed to be correlated with the aggressiveness of the *P. capsici* isolate. Bnejdi *et al.* (2009) reported that the probability of goodness-of-fit of models was negatively correlated with the aggressiveness of the *P*. *nicotianae* isolate*.* Both types of resistance to different isolates of *P. palmivora* were reported by Surujdeo-Maharaj *et al.* (2001). Generation means analysis is the methodology generally used to study quantitative trait inheritance, including interaction between non-allelic genes (Mather and Jinks, 1974).

The objective of the present study was to investigate the types of gene action governing the inheritance of resistance to different aggressiveness of *P.nicotianae* isolates in pepper.

## Materials and Methods

###  Plant material

This study was carried out at the National Institute for Agricultural Research in Tunis, Tunisia. Pepper (*C. annuum* L.) parental lines were selected based on their resistance to *P.nicotianae*. The resistant parent (Pr) used was cv. CM334 and the susceptible parents (Ps) were cvs. Beldi and Nabeul II. Crosses were made as follows: CM334 x Beldi, and CM334 x Nabeul II. Generation means analysis was performed using each of the Pr and Ps, F_1_ and F_2_ generations, and backcrosses of F_1_ to each parent (BC_1_ Pr and BC_2_ Ps). All crosses were controlled pollinations in a greenhouse.

###  Inoculum preparation

Six *P. nicotianae* isolates were collected from infected pepper plants from three different regions in Tunisia: Pn_Ko1_ and Pn_Ko2_ from Korba, Pn_Bz1_ and Pn_Bz2_ from Bizert, and Pn_Kr1_ and Pn_Kr2_ from Kairown. These isolates were identified as *P. nicotianae* according to morphological and biological characteristics described by Allagui *et al.* (1995) and Allagui and Lepoivre (2000).

###  Pepper seeding and inoculation

Pepper seeds were surface-sterilized with 4% sodium hypochlorite, rinsed twice with distilled water and dried on filter paper. The substrate used for sowing and planting was a mixture of clay soil, sand and peat (2:1:1, v/v/v). Two weeks after sowing, the seedlings (two-cotyledon stage) were transplanted into alveolated plates containing the same substrate disinfected by heat. Plants were grown in a randomized complete block design, with two replications. Two weeks after transplantation, seedlings (two-leaf stage) from each replication were inoculated with different isolates by dripping a suspension of 280,000 zoospores (in 3.5 mL) onto the collar of each plant. Control and inoculated plants were maintained in a greenhouse at 28-30 °C. Plants were irrigated with tap water every 3-4 d.

###  Assessment of root necrosis

After three weeks of incubation, the root system of each seedling was delicately detached from the substrate by washing in a water bowl. Root necrosis intensity was evaluated according to the following scale: 0 (healthy plant), 0.5 (necrosis limited to the extremity of radicles), 1 (necrosis on the lower half of primary roots), 2 (necrosis all over the primary roots), 3 (necrosis reaching the crown and the lateral roots), 4 (hypocotyl rotten), and 5 (whole plant dead). The number of plants evaluated varied depending on the generation and was greater in generations with greater segregation, such as F_2_, BC_1_P_r_, and BC_1_P_s_. Prior to analysis, transforming the data by log, square root, arc-sine and arc-sine of square root of the variable had no effect on data distribution or in removing epistatic effects.

###  Statistical analyses

Analysis of variance by population and isolate using GLM procedures (SAS, 1990) indicated that the replication and generation x replication effects were not significant. Therefore, generation means analysis was conducted without adjusting data for replication.

###  Gene effects

Weighted least squares regression analyses were used to solve for mid-parent [m] pooled additive [d], pooled dominance [h] and pooled digenic epistatic ([i], [l] and [j]) genetic effects, following the models and assumptions described in Mather and Jinks (1982). A simple additive-dominance genetic model containing only the m, d and h effects was tested first, using the joint scaling test described in Rowe and Alexander (1980). Adequacy of the genetic model was assessed using a chi-square goodness-of-fit statistic derived from deviations from this model. If statistically significant at p < 0.05, the genetic models containing digenic epistatic effects were then tested until the chi-square statistic was non-significant.

###  Heritability and gene number

Homogeneity of variances of non-segregating generations was tested using Bartlett's test (Bartlett, 1937), and whenever the variances were heterogeneous the environmental variance (*V*_*E*_) was replaced by an adequate number of separate parameters and pooled to produce a single environmental variance. Additive, dominance and environmental variance components were estimated using the maximum likelihood method, with the observed variance of the six basic generations being used as the initial weights (df/2 x S^2^) until the chi-square test value reached a minimum (Lynch and Walsh, 1998).

Narrow-sense heritability (h^2^_n_) was calculated according to the formula: h^2^_n_ = [V*_A_/(V*_A_+ V*_D_*+ V*_*E*_)], where V*_A_ is the additive genetic component of variance, V*_D_ the dominance genetic component of variance, and *V*_*E*_ the environmental component of variance (Kearsey and Pooni, 1996). The V*_D_ value was set to zero when the estimated variance turned out to be negative.

The number of genes contributing to resistance was estimated by the method of Lande (1981), as follows: N = (P_1_ - P_2_)^2^ [1.5-2h (1 - h)]/ 8[σ^2^_F2_ - 0.25 (σ^2^_P1_ + σ^2^_P2_ + 2 σ^2^_F1_)], where h = (F_1_ -P_1_) / (P_2_ - P_1_). This method assumes that loci are not linked, alleles are of equal effect, genes with positive and negative influence are fixed in alternate lines and, most critically, alleles have an additive effect on phenotype. Violation of one or more of these assumptions will generally lead to an underestimate of the number of effective factors (Zeng *et al.*, 1990; Zeng, 1992).

## Results

**Figure 1:**
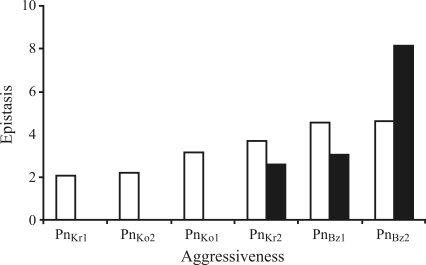
Mean of aggressiveness of six *P. nicotianae* isolates revealed in cv.  Beldi and absolute total of epistasis in cross Beldi x CM344.  Column of aggressiveness followed by the same letter is not significantly different at p < 0.05. ■ Epistasis (measured as fellow: epistasis = |i| + |l| + |j|). □ Aggressiveness (means of necrosis revealed in the susceptible parent Beldi).

**Figure 2:**
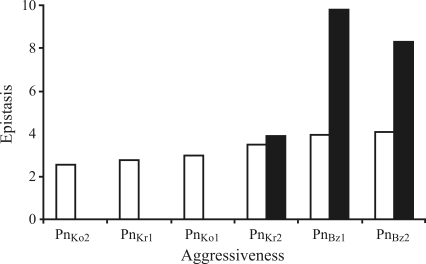
Mean of aggressiveness of six *P. nicotianae* isolates revealed in cv. Nabeul II and absolute total of epistasis in cross Nabeul II x CM344. Column of aggressiveness followed by the same letter is not significantly different at p < 0.05. ■ Epistasis (measured as fellow: epistasis = |i| + |l| + |j|). □ Aggressiveness (means of necrosis revealed in the susceptible parent Nabeul II).

Parental means and their standard errors for both crosses are given in Table 1. In all cases, depending on the isolates, the means of the parents in each cross tended to be more extreme. The means of backcrosses BC_1_P_r_ and BC_1_P_s_ tended to be close to those of their respective recurrent parents. These results confirmed the choice of parents for the present study. Different classes of aggressiveness were detected for the six *P.nicotianae* isolates (Figures 1  and 2).

Generation means analysis is shown in Table 2. For isolates Pn_Ko1_, Pn_Ko2_ and Pn_Kr1_, the three-parameter model was sufficient to explain the inheritance of resistance to *P.nicotianae* for the two crosses. The additive effect was significant and negative in all cases. The dominance effect was negative in the majority of cases and significant in only three cases.

For isolates Pn_Bz1_, Pn_Bz2_ and Pn_Kr2_, the three-parameter model failed to explain variation in generation means in the two crosses. Therefore, a digenic epistatic model was applied and found adequate in two cases for the cross Beldi x CM 334, and in one case for Nabeul II x CM 334. For the cross Beldi x CM 334 and isolate Pn_Bz2_, and the combinations of cross Nabeul II x CM 334 and isolates Pn_Bz1_ and Pn_Bz2_, both models failed to explain the variation in generation means.

With regard to epistatic effects, the additive x additive effect was significant in five cases and negative in four cases. The additive x dominance (j) and dominance x dominance (l) effects in all cases were more important than the additive x additive (i) effects for the cross Nabeul II x CM 334. For the combinations of isolates Pn_Bz2_ with Beldi x CM 334, and Pn_Bz1_ and Pn_Bz2_ with Nabeul II x CM 334, both models failed to explain the variation between generations, with all three epistatic components being significant. When epistasis was detected, the total of absolute epistatic effects increased when aggressiveness increased (Figures 1  and 2).

Variance components were estimated and used to calculate h^2^_n_ for both crosses and six isolates. Additive variance was positive and of greater magnitude than environmental variance in all cases. In the two crosses, for isolates Pn_Ko1_, Pn_Ko2_ and Pn_Kr1_, heritability averaged 0.976, with a range of 0.95-0.99. For isolates, Pn_Bz1_, Pn_Bz2_ and Pn_Kr2_, heritability averaged 0.701, with a range of 0.27-0.96. The number of genes varied between 0.78 and 1.48 for Beldi x CM 334 and between -3.74 and 2.71 for the Nabeul II x CM 334 cross (Table 3).

## Discussion

There were significant differences among generation means in all cases, revealing genetic diversity for this attribute in the materials studied, thus validating the genetic analysis of the traits according to the method of Mather and Jinks (1982).

Although varying with the cross and the class of aggressiveness of the isolates, the variation in generation means fitted an additive dominance model for Pn_Ko1_, Pn_Ko2_ and Pn_Kr1_ in the two crosses. The additive effect was significant and greater than the dominance effect. The fact that the additive and dominance effects were negative indicated that they contributed more to resistance than to susceptibility.

For Pn_Bz1_, Pn_Bz2_ and Pn_Kr2_, the digenic epistatic model was adequate in three cases. In the other cases, none of the models explained the variation between generations, indicating more complex mechanisms of genetic control. To identify whether the model failure was due to higher-order interactions or linkage effects, further analyses of sufficient generations to fit a full trigenic interaction and linkage model should be performed. Generation means analysis indicated that the comportment of the two crosses for resistance to different isolates was similar. For the isolates with aggressiveness levels of 2.05-3.16, an additive-dominance model was fitted. For the isolates with level of aggressiveness = 3.63, the epistatic effect was an integral component of resistance to *P. nicotianae*, and the aggressiveness level determined the epistasis. For the *P. nicotianae* isolates of greater aggressiveness, the pepper presumably developed more mechanisms and solicited more interactions for resistance. For the less aggressive isolates Pn_Ko1_, Pn_Ko2_ and Pn_Kr1_, the epistasis of resistance was not induced. The development of absolute totals of epistatic effects and aggressiveness of isolates confirmed this result (Figures 1  and 2). Bartual *et al.* (1991) found that epistasis was a principal source of variation in resistance of pepper to the *Phytophthora* stem blight, and was correlated with the level of pathogen aggressiveness. In the present study, for the less aggressive isolates, only additive and dominance models were applied and found sufficient. With high levels of aggressiveness, the additive and dominance effects were not sufficient to explain variation in generation means, and the pepper plants developed more mechanisms of resistance to *P*. *nicotianae*, such as epistasis. Selection with less aggressive isolates was efficient, but not with more aggressive isolates. On the other hand, selection with more aggressive isolates was more stable than with less aggressive isolates.

Correct estimates of the number of genes contributing to genetic variation of quantitative characters within and between populations are fundamental to quantitative genetics (Zeng, 1992). Gene numbers based on the formula of Lande (1981) are conservative, expressing the minimum number of genes controlling a character. The number of genes controlling resistance to *P. nicotianae* was underestimated in the two crosses for isolates Pn_Bz1_, Pn_Bz2_ and Pn_Kr2_, due to the failure to meet the analysis assumptions of no epistasis and no dominance, since some dominance and epistatic effects were significant. For isolate Pn_Ko1_, epistasis was absent and dominance was not significant in the two crosses (the best case). Thus the actual number of genes averaged 2, with a range of 1.32-2.71. Irzhansky and Cohen (2006) found two genes for resistance to *P. infestans* in *Lycopersicon pimpenellifolium*.

The values of narrow-sense heritability (h^2^_n_) varied depending on the isolates tested. In all cases, heritability was higher for isolates with lower aggressiveness, and moderate to high for isolates with higher aggressiveness. For the plant breeder, h^2^_n_ is important, as the effectiveness of selection depends on the additive portion of genetic variation in relation to total variance (Falconer, 1960). In the present study, moderate to high values for h^2^_n_ suggested that genetics had a considerable participation in the phenotypic expression of traits and that selection for the traits should be efficient.

## Figures and Tables

**Table 1:** Root necrosis means ± SE (x 100) for six *P. nicotianae* isolates in parents and offspring populations from two pepper crosses of susceptible (s) x resistant (r) parents.

Population	Pn_Ko1_	Pn_Ko2_	Pn_Bz1_	Pn_Bz2_	Pn_Kr1_	Pn_Kr2_
Beldi (s) x CM 334 (r)						
P_s_	3.16 ± 1.41 a (9)	2.22 ± 0.67a (9)	4.56 ± 0.53a (10)	4.67 ± 0.5a (10)	2.06 ± 0.88a (10)	3.67 ± 1.32a (10)
BC_1_P_s_	1.93 ± 1.6 b (56)	1.65 ± 1.12b (50)	2.31 ± 1.7b (60)	2.37 ± 1.66bc (40)	1.58 ± 1.53ab (52)	0.25 ± 0.26b (50)
F_1_	0.57 ± 0.32 c (50)	1.23 ± 1.07bc (30)	1.82 ± 1.45bc (60)	1.86 ± 1.13c (50)	1.13 ± 1.02bc (43)	0.65 ± 0.53de (60)
F_2_	1.25 ± 1.48 bc (360)	1.03 ± 1.06cd (300)	1.81 ± 1.64bc (120)	3.00 ± 1.69b (260)	1.23 ± 1.09bc (240)	1.61 ± 1.37bc (150)
BC_1_P_r_	0.58 ± 0.28 c (50)	0.51 ± 0.32ed (50)	0.94 ± 1.26cd (50)	1.64 ± 1.11c (50)	0.67 ± 0.50dc (50)	2.11 ± 1.62cd (50)
P_r_	0.45 ± 0.15 c (10)	0.10 ± 0.21e (10)	0.45 ± 0.37d (10)	0.60 ± 0.39d (10)	0.15 ± 0.24d (10)	1.10 ± 0.05e (10)

Nabeul II (s) x CM 334 (r)						
P_s_	3.07 ± 2.89a (15)	2.67 ± 1.05 a (15)	4.07 ± 0.88a (15)	4.27 ± 0.96a (15)	2.87 ± 1.25a (15)	3.63 ± 1.56a (15)
BC_1_P_s_	2.03 ± 1.41b (52)	2.11 ± 1.55ab (53)	1.39 ± 1.57c (60)	2.10 ± 1.35bc (43)	2.29 ± 1.85ab (55)	2.28 ± 1.45b (54)
F_1_	0.97 ± 2.24cd (43)	1.81 ± 1.52 b (40)	1.23 ± 1.36c (63)	1.70 ± 1.51c (53)	1.71 ± 1.36b (57)	0.76 ± 0.60c (67)
F_2_	1.34 ± 1.58c (289)	1.48 ± 1.33b (220)	2.14 ± 1.85b (203)	2.69 ± 1.67b (260)	1.83 ± 1.74b (209)	2.30 ± 1.57b (205)
BC_1_P_r_	0.65 ± 0.37d (42)	0.78 ± 0.65c (46)	1.44 ± 1.26c (45)	1.99 ± .48bc (45)	0.96 ± 0.88c (50)	1.62 ± 1.47b (49)
P_r_	0.45 ± 0.03d (10)	0.10 ± 0.21d (10)	0.45 ± 0.37d (10)	0.60 ± 0.39d (10)	0.15 ± 0.24d (10)	0.25 ± 0.26c (10)

Means followed by the same letter within each column were not significantly different at p < 0.05. Numbers of plants evaluated in each generation are shown in parenthesis.

**Table 2:** Estimates of gene effects ± SE (x 100) for pepper resistance to six *P. nicotianae* isolates in two crosses of susceptible (s) x resistant (r) parents.

Model	Pn_Ko1_	Pn_Ko2_	Pn_Bz1_	Pn_Bz2_	Pn_Kr1_	Pn_Kr2_
Beldi (s) x CM 334 (r)						
Three-parameter model						
m	1.87 ± 10**	1.13 ± 7**	2.31 ± 9**	2.68 ± 9**	1.16 ± 9**	2.12 ± 10**
d	-1.36 ± 9**	-1.08 ± 6**	-1.85 ± 8**	-1.8 ± 8**	-1.0 ± 8**	-1.6 ± 10**
h	-1.25 ± 11**	-0.14 ± 13	-0.97 ± 19**	-0.5 ± 17**	0.03 ± 16**	-1.2 ± 14**
(P)	0.36	0.29	< 0.001	< 0.001	0.58	< 0.001

Best fit model						
m			2.50 ± 10**	15.5 ± 49**		1.95 ± 22**
d			-2.05 ± 10**	-2.0 ± 10**		-1.70 ± 22**
h			-2.46 ± 44**	-9.7 ± 125**		-0.08 ± 70**
i				-4.0 ± 48**		1.38 ± 52**
l			1.77 ± 0.52**	4.95 ± 83**		-1.22 ± 51**
j			1.26 ± 0.39**	2.59 ± 38**		-
(P)			0.14	-		0.92

Nabeul II (s) x CM 334 (r)						

Three-parameter model						
m	1.80 ± 9**	1.39 ± 10**	1.76 ± 10**	2.05 ± 11**	1.57 ± 12**	2.26 ± 9**
d	-1.36 ± 9**	-1.31 ± 9**	-0.88 ± 8**	-0.96 ± 10**	-1.41 ± 11**	-1.7 ± 9**
h	-0.93 ± 7**	0.13 ± 17	-0.39 ± 19**	0.13 ± 22	0.21 ± 19	-1.27 ± 13**
(P)	0.96	0.59	< 0.001	< 0.001	0.56	< 0.001

Best fit model						
m			17.58 ± 58**	6.46 ± 50**		3.94 ± 21**
d			-1.80 ± 12**	-1.80 ± 13**		-1.79 ± 18**
h			-8.13 ± 134**	-5.92 ± 123**		-3.16 ± 25**
i			-2.89 ± 57**	-2.55 ± 49**		-1.89 ± 30**
l			4.2 ± 83**	2.63 ± 83**		-
j			3.72 ± 35**	3.44 ± 38**		2.17 ± 51**
(P)			-	-		0.25

Mean (m), additive (d), dominance (h), additive x additive (i), additive x dominance (j) dominance x dominance (l) genetic effects for the model. y = m + d + h + i + j + l, where y is the generation mean. (P): Probability of adequateness of model. *,** indicates means and gene effects are statistically different from zero at p < 0.05 and p < 0.01, respectively.

**Table 3:** Estimates of additive (V*_A_), dominance (V*_D_) and environmental variances (*V*_*E*_) with ± SE (x 100), narrow-sense heritabilities (h^2^_n_) and minimum number (*N*) of genes (or effective factors) for resistance to six isolates of *P.nicotianae* in two pepper crosses of susceptible (s) x resistant (r) parents.

	Pn_Ko1_	Pn_Ko2_	Pn_Bz1_	Pn_Bz2_	Pn_Kr1_	Pn_Kr2_
Beldi (s) x CM 334 (r)						
*V*_*E*_	0.04 ± 0.7*	0.05 ± 1*	0.19 ± 3*	0.23 ± 4*	0.07 ± 1*	0.12 ± 2*
V*_A_	4.26 ± 33*	2.01 ± 16*	1.56 ± 50	2.69 ± 48*	1.80 ± 18	3.73 ± 28
V*_D_	-2.09 ± 16*	-0.94 ± 8.5	0.91 ± 36*	-0.09 ± 32*	-0.71 ± 10*	-1.98 ± 14*
*X*^*2*^*(df)*	(3)ns	(3)ns	(3)ns	(3)ns	(3)ns	(3)ns
h^2^_n_	0 .99	0.97	0.58	0.91	0.95	0.96
*(N)*	0.78	1.32	1.46	1.05	1.01	1.48

Nabeul II (s) x CM 334 (r)						

*V*_*E*_	0.02 ± 0.4*	0.05 ± 0.9*	0.21 ± 03*	0.23 ± 0.4*	0.07 ± 01*	0.12 ± 1*
V*_A_	2.22 ± 21*	2.60 ± 34	3.05 ± 71	1.68 ± 55*	4.42 ± 60*	0.67 ± 58
V*_D_	-0.66 ± 12	-0.90 ± 18*	0.14 ± 41	0.88 ± 35*	-1.45 ± 32*	1.66 ± 39*
*X*^*2*^*(df)*	(3)ns	(3)ns	(3)ns	(3)ns	(3)ns	(3)ns
h^2^_n_	0.98	0.98	0.89	0.60	0.98	0.27
*(N)*	-3.74	2.71	0.84	1.3	0.54	1.07

df = degrees of freedom, calculated as the number of generations minus the number of estimated variance parameters. ns = non-significant.
